# Tertiary Lymphoid Structures Are Associated with Favorable Clinical Outcomes and Negatively Correlated with Cancer-Associated Fibroblasts in Esophageal Cancer

**DOI:** 10.3390/cancers17203351

**Published:** 2025-10-17

**Authors:** Tomoyoshi Kunitomo, Kazuhiro Noma, Noriyuki Nishiwaki, Seitaro Nishimura, Yasushige Takeda, Hijiri Matsumoto, Tatsuya Takahashi, Kento Kawasaki, Masaaki Akai, Naoaki Maeda, Satoru Kikuchi, Shunsuke Tanabe, Toshiaki Ohara, Hiroshi Tazawa, Yasuhiro Shirakawa, Toshiyoshi Fujiwara

**Affiliations:** 1 Department of Gastroenterological Surgery, Graduate School of Medicine, Dentistry and Pharmaceutical Sciences, Okayama University, Okayama 700-8558, Japan; 2 Department of Pathology & Experimental Medicine, Graduate School of Medicine, Dentistry and Pharmaceutical Sciences, Okayama University, Okayama 700-8558, Japan; 3 Center for Innovative Clinical Medicine, Okayama University Hospital, Okayama 700-8558, Japan; 4 Department of Surgery, Hiroshima City Hiroshima Citizens Hospital, Hiroshima 730-8518, Japan

**Keywords:** tertiary lymphoid structures (TLSs), cancer-associated fibroblasts (CAFs), esophageal cancer, tumor microenvironment, prognosis

## Abstract

Background: Esophageal cancer remains a highly aggressive malignant tumor with poor prognosis, despite advances in combination therapies and novel immunotherapies. Tertiary lymphoid structures (TLSs), characterized by densely packed CD20^+^ B cells in a germinal-center-like structure, have recently been recognized as immune-stimulating components within the tumor microenvironment. In contrast, cancer-associated fibroblasts (CAFs) are stromal cells expressing fibroblast-activating protein (FAP) involved in immunosuppression. Methods: In this retrospective study, 124 clinical samples from patients who underwent radical surgery for esophageal cancer at our institute were analyzed. We investigated whether TLSs could serve as a prognostic factor and examined their association with tumor microenvironment factors. Results: The presence of TLSs was an independent prognostic factor for overall and progression-free survival in multivariate analyses. A high level of TLS formation correlated with better nutritional status, fewer M2 macrophages, and greater plasma cell infiltration. Additionally, little TLS formation was observed in areas with abundant CAFs, and quantitative analyses revealed a significant negative correlation between TLSs and CAFs. Conclusions: TLSs enhance antitumor immunity via macrophages and plasma cells and can be a valuable prognostic indicator in patients undergoing surgery for esophageal cancer. Targeting CAFs may prove to be a promising therapeutic strategy to enhance tumor-immunity-related TLSs.

## Simple Summary

Tertiary lymphoid structures (TLSs) are immune cell aggregates that can develop within tumors. In esophageal cancer, we found that TLSs are present and associated with better patient outcomes. We also observed that cancer-associated fibroblasts (CAFs), which are important components of the tumor microenvironment, are related to TLS formation. These findings suggest that CAFs may influence the development of TLSs. Therapies targeting CAFs could therefore enhance TLS formation and potentially improve prognosis in patients with esophageal cancer.

## 1. Introduction

Notable progress has recently been made in the use of immune checkpoint inhibitors for the treatment of malignancies, including esophageal cancer, which is largely classified into two types—squamous cell carcinoma and adenocarcinoma. Initially, nivolumab and pembrolizumab, which are anti-programmed cell death 1 (PD-1) antibodies, were effective as second-line therapies for patients with unresectable advanced or recurrent esophageal cancer [1,2]. Currently, treatments that are Food and Drug Administration (FDA)-approved for clinical use and are widely used in clinical practice worldwide include pembrolizumab in combination with a dual chemotherapy regimen as a first-line treatment [3]; nivolumab as an adjuvant therapy for patients who underwent surgery after preoperative chemoradiotherapy [4]; and nivolumab combined with ipilimumab, an anti-cytotoxic T lymphocyte-associated protein 4 (CTLA-4) antibody [5]. These findings strongly suggest that an immune-mediated mechanism is an important therapeutic target in esophageal cancer. However, while overall efficacy has been demonstrated, subgroup analyses indicate potential variations in response among different patient populations. For instance, differences in efficacy may exist depending on histological subtype, highlighting the ongoing controversy surrounding these treatments [2,3].

The advent of immunotherapy has prompted increased interest in targeting the tumor microenvironment (TME). Tertiary lymphoid structures (TLSs) are found in tissues subjected to chronic inflammation and antigen persistence in patients with autoimmune diseases, chronic infections, graft rejection, and cancers [6,7]. In many cancers, all major B-cell subsets, from naive B cells to plasma cells, can be found in the TME [8]. B-cell clones differentiate into plasma cells that produce immunoglobulin G (IgG) or IgA antibodies that target tumor-associated antigens [8]. B cells differentiate and mature in the TLS, and mature plasma cells invade the tumor and contribute to tumor immunity [9]. Therefore, TLSs have potential antitumor effects via B-cell-based humoral immunity [10]. Additionally, TLSs are associated with a good prognosis in colon, lung [11,12], and pancreatic cancers [13] and with good responses to immunotherapy in melanoma [14,15], bone and soft tissue sarcoma [16], and renal cell carcinoma [14]. The presence of mature TLSs with CD23^+^ dendritic cells is a predictor of the response to immune checkpoint inhibitors [17]. Thus, TLSs are a potential biomarker for immunotherapy.

As another critical TME component, cancer-associated fibroblasts (CAFs) play pivotal roles in cancer growth, metastasis, migration, invasion, and chemoresistance [18,19]. Using fibroblast activation protein (FAP) as a CAF marker, we previously revealed that FAP^+^ CAFs are linked to poor survival in patients with esophageal cancer [19,20]. In tumor immunosuppression, CAFs suppress host tumor immunity in vivo and in clinical practice; increased CD8^+^ tumor-infiltrating lymphocytes (TILs) and decreased forkhead box P3 (FoxP3)^+^ TILs are significantly correlated with CAFs and outcomes [20]. CAFs were reported to affect the B-cell-mediated formation of TLSs. While CAFs can express lymphoid tissue organizer markers such as VCAM-1 and ICAM-1 and produce the B-cell organizer chemokine CXCL13, FAP^+^ CAFs have been reported to have less lymphoid tissue organizer capability in vivo [21]. However, the association between FAP^+^ CAFs and TLSs in human tumor tissues is unclear.

In this study, we investigated the prognostic value of TLSs in esophageal cancer and their correlation with CAFs using clinical specimens and bioinformatics data.

## 2. Materials and Methods

### 2.1. Patients and Tissue Samples

In total, 149 patients who underwent radical surgery for esophageal cancer at the Department of Gastroenterological Surgery of Okayama University Hospital between 2008 and 2010 were identified. Patients were excluded if they had undergone endoscopic mucosal resection (EMR), endoscopic submucosal dissection (ESD), or preoperative radiotherapy or chemoradiotherapy followed by surgery; were diagnosed with melanoma or distant metastasis; or showed tumor absence/complete response after neoadjuvant therapy. Patients were also considered ineligible if they presented with high fever at the time of surgery, uncontrolled diabetes mellitus or hypertension, recent unstable cardiovascular disease, or severe pulmonary conditions such as uncontrolled interstitial pneumonia or advanced emphysema that would increase surgical risk. However, none of the patients in our cohort met these severe comorbidity exclusion criteria during the study period. A total of 124 patients were enrolled in this study. Data on age, sex, histological type, neoadjuvant therapy, pathological invasion depth (pT), and lymph node status (pN) were reviewed for all patients. Tumors were classified according to the TNM Classification of Malignant Tumors, 7th edition (UICC, 7th edition).

Preoperative blood test results were obtained on admission for surgery. The lymphocyte-to-monocyte ratio (LMR) was calculated by dividing the number of lymphocytes (/mm^3^) by the number of monocytes (/mm^3^) [22,23]. The prognostic nutritional index (PNI) was calculated as 10 × serum albumin (g/dL) + 0.005 × number of lymphocytes (/mm^3^) [24].

### 2.2. Antibodies

The following antibodies were used in this study: monoclonal mouse anti-CD20 (Leica Biosystems, Wetzlar, Germany; clone L26; 1:150 dilution) for immunohistochemical analyses (IHC) and immunofluorescence staining (IF); monoclonal mouse anti-human CD3 (Dako, Glostrup, Denmark; clone F7.2.38; 1:50 dilution) for IHC and IF; monoclonal mouse anti-human CD8 (Dako; clone C8/144B; 1:100 dilution) for IHC; monoclonal mouse anti-human multiple myeloma oncogene-1 (MUM-1) protein (Dako; clone MUM1p; 1:50 dilution) for IHC and IF; monoclonal rat anti-peripheral node addressin (MECA-79; NOVUS, Centennial, CO, USA; catalog number NB100-77673; 1:100 dilution) for IF; monoclonal rabbit anti-ionized calcium-binding adapter molecule 1 (Iba1) (Abcam, Cambridge, UK; catalog number ab178846; 1:2000 dilution) for IHC; monoclonal rabbit anti-CD163 (Abcam, Cambridge, UK; catalog number ab182422; 1:100 dilution) for IHC and IF; monoclonal mouse anti-FoxP3 (Abcam; catalog number ab20034; 1:100 dilution) for IHC and IF; monoclonal mouse anti-α-smooth muscle actin (αSMA) (Sigma, St. Louis, MO, USA; code A2547; clone 1A4; 1:1000 dilution) for IHC and IF; and monoclonal rabbit anti-FAP alpha (Abcam; catalog number ab207178; 1:250 dilution) for IHC and IF.

### 2.3. Immunohistochemical Analysis

Tissue blocks of formalin-fixed and paraffin-embedded surgical specimens of esophageal cancer tissues were sectioned into 3 μm slices for IHC. First, the presence of tumors was confirmed using hematoxylin and eosin (HE) staining. Sectioned tissues were deparaffinized and soaked in 0.3% H_2_O_2_ in methanol at room temperature (RT) for 10 min to eliminate endogenous peroxidase activity. Antigen retrieval was performed by heating specimens in ethylenediaminetetraacetic acid (EDTA) buffer in a microwave. After cooling, the sections were incubated in peroxidase blocking reagent (Dako) for 10 min at RT. The sectioned tissues were incubated with primary antibodies against CD20, CD8, FoxP3, Iba1, CD163, αSMA, and FAP for 30 min at RT or against MUM-1 overnight at 4 °C. Following three 5 min washes with PBS, the sections were incubated with anti-mouse/rabbit secondary antibodies (Dako, EnVision + System-HRP Labeled Polymer) for 30 min at RT. After washing, the enzyme substrate 3,3′-diaminobenzidine (Dako) was used for visualization, and the sections were counterstained with Mayer’s hematoxylin.

### 2.4. Quantitation of TLS, Tumor-Infiltrating Lymphocytes, and the FAP Area Index

First, tumor boundaries were confirmed by HE staining under a microscope at low magnification (4× objective lens). Four non-overlapping fields with abundant circular CD20^+^ clusters were selected and photographed. CD20^+^ clusters were counted. Four non-overlapping fields at high magnification (40× objective lens) with abundant MUM-1^+^ cells, CD8^+^ and FoxP3^+^ TILs, and Iba1^+^ and CD163^+^ macrophages were selected and imaged. TILs and macrophages were counted using ImageJ software version 1.53 (https://imagej.net/ij/). CAFs were defined as fibroblasts expressing FAP, and two FAP scoring systems were used. For the first scoring system, the area index was calculated using ImageJ. The second scoring system was the sum of the proportion (0, no staining; 1, <10% staining; 2, <30%; 3, <60%; and 4, ≥60%) and intensity scores (0, none; 1, weak; 2, intermediate; and 3, strong), as previously reported [19]. All cases were divided into three groups according to the FAP score: ≥5, high; 2–4, moderate; and 0 or 1, weak.

### 2.5. Double Immunohistochemistry

Double immunohistochemical staining was performed to evaluate the association between FAP and TLSs. For double immunostaining, the sections were stained with one of the stains, heated in a microwave, and then subjected to the second staining procedure. The sections were then incubated with the second set of primary antibodies, followed by incubation with the secondary antibodies (Dako, EnVision + System-HRP Labeled Polymer). A HistoGreen Peroxidase Substrate Kit (Eurobio Ingen, Chilly-Mazarin, France) was used for the second detection reaction.

### 2.6. Immunofluorescence Staining

Slides treated with the secondary antibodies were incubated with the appropriate Opal fluorophores for 10 min in the dark and then washed with Tris-Buffered Saline with Tween 20 (TBS-T). Multiplex staining was performed by removing the antibody complex via microwave incubation, and the staining cycles were repeated. After staining, the slides were counterstained with NucBlue (Invitrogen, Waltham, MA, USA) for 5 min at RT, washed with TBS-T, and covered with coverslips. The stained slides were observed, and the images were captured using an all-in-one fluorescence microscope (Keyence, Osaka, Japan).

### 2.7. Bioinformatic Datasets

Data from The Cancer Genome Atlas (TCGA) were acquired using the TCGAbiolinks package (version 2.22.4) in R. RNA-seq data were quantile-normalized and transformed to log2 (data + 1). The TLS signature score was calculated as the mean gene expression level. TLS-hallmark genes (*CCL19*, *CCL21*, *CXCL13*, *CCR7*, *CXCR5*, *SELL*, and *LAMP3*) were selected for analyses based on a previous report [8]. For survival analyses, the signature scores were divided into two groups: the top 25% and the remaining 75%. Overall survival (OS) and progression-free survival (PFS) were determined as described in previous reports [25].

### 2.8. Statistical Analyses

All statistical analyses were performed using EZR (Saitama Medical Center, Jichi Medical University, Saitama, Japan) [26]. EZR is a modified version of R commander designed to add statistical functions frequently used in biostatistics and a graphical user interface for R version 4.0.5 (The R Foundation for Statistical Computing, Vienna, Austria). OS and PFS were defined from the date of surgery to death from any cause, with PFS additionally including recurrence. Patients were censored at the date of last confirmed survival. The correlations between TLSs and clinicopathological features were examined using Fisher’s exact test and the Mann–Whitney U test, as appropriate, and Spearman’s rank correlation coefficients were calculated. Cumulative survival was evaluated using the Kaplan–Meier method, and the survival proportions were compared using the log-rank test. The Cox proportional hazards model was used for univariate and multivariate analyses. The hazard ratios (HRs) and 95% confidence intervals (CIs) for the clinical and pathological factors were calculated using Cox proportional hazards regression in univariate and multivariate analyses. A multivariate analysis was performed using a stepwise method based on the Akaike information criterion (AIC) to extract and examine the factors.

## 3. Results

### 3.1. Identification of TLSs and Their Prognostic Value in Esophageal Cancer

**Figure 1:**
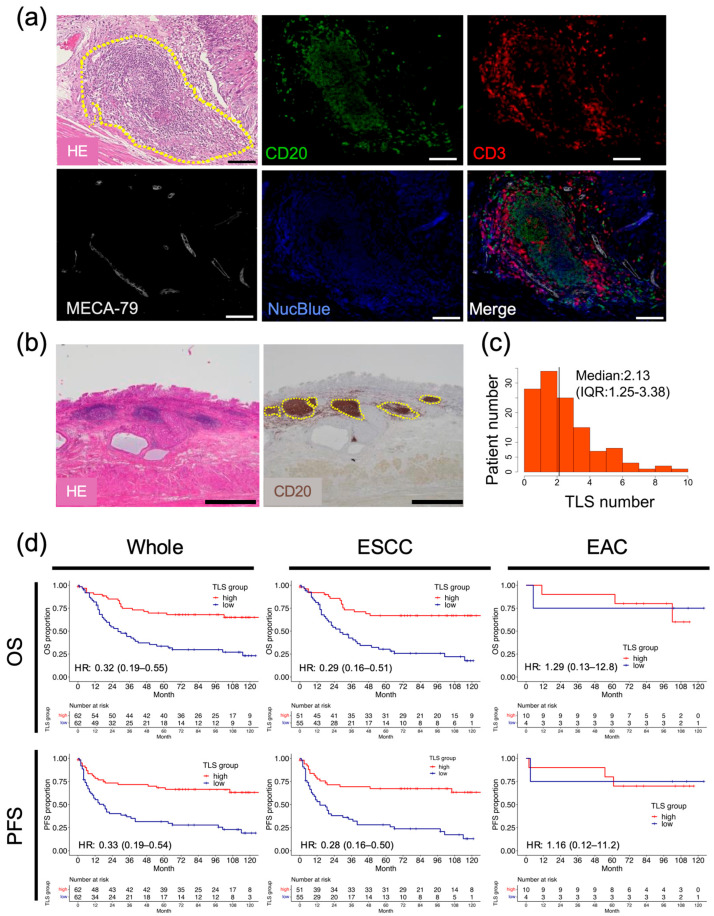
Association between the presence of TLSs in esophageal cancer specimens and prognosis. (**a**) Representative images showing HE-stained and multiplex immunofluorescence-stained TLSs in esophageal cancer specimens. The yellow dashed circle indicates a TLS. CD20 (green), CD3 (red), MECA-79 (white), and NucBlue denote B cells, T cells, HEVs, and the nucleus, respectively. Scale bars: 100 µm. (**b**) Representative images showing HE staining and IHC of CD20-expressing cells in patients with esophageal cancer. Dotted lines show the borders of TLSs. Scale bars: 1000 µm. (**c**) Histogram of TLS counts in all patients (black bar, median value). (**d**) Survival analyses by OS and PFS, conducted using our institutional data, according to histological factors. Whole: *n* = 124, ESCC: *n* = 106, EAC: *n* = 14 (Cox regression hazard model; HR, hazard ratio with 95% confidence intervals).

To confirm the presence of TLSs in esophageal cancer tissues, HE staining and multiplex immunofluorescence imaging were performed using resected esophageal cancer specimens (Figure 1a). These images revealed clusters of lymphocytes within the tumor, which were composed of CD3^+^ or CD20^+^ lymphocytes, indicating TLS formation by T and B cells [27]. MECA-79-positive cells were also detected in the clusters, suggesting that high endothelial venules (HEVs) are components of TLSs [28]. B cells are the predominant cellular component of TLSs found in most other types of cancers and are often associated with favorable clinical outcomes [27]. We assessed clusters of CD20^+^ B cells using IHC to quantify TLSs in the surgical specimens (Figure 1b). TLSs were detected in 112/124 (90.3%) surgically resected specimens, and the median number of TLSs per high-magnification field was 2.13 (interquartile range [IQR]: 1.25–3.38; Figure 1c). To investigate the associations between TLSs and outcomes in patients with esophageal cancer, the clinicopathological parameters were compared between two groups: TLS^high^ and TLS^low^ groups. The background characteristics of each group are shown in Supplementary Table S1. Age, sex, height, weight, neoadjuvant therapy, histological type, location of the primary tumor, pathological tumor depth, lymph node metastasis, and staging did not differ significantly between the two groups. Regarding the long-term prognosis, both postoperative OS and PFS were significantly higher in the TLS^high^ group than in the TLS^low^ group (Figure 1d; OS, HR = 0.32, 95% CI = 0.19–0.55, *p* < 0.001; PFS, HR = 0.33, 95% CI = 0.19–0.54, *p* < 0.001). Next, we performed subgroup analyses according to histological features. In the histologically based subgroup analysis of survival, the TLS^high^ group in esophageal squamous cell carcinoma (ESCC) (*n* = 106) had significantly better PFS and OS than the TLS^low^ group, while no significant relationships were observed in esophageal adenocarcinoma (EAC; *n* = 14). In our cohort, a multivariate analysis revealed that a high level of TLS formation was an independent prognostic factor for OS and PFS in all cases and in ESCC (Supplementary Table S2).

To validate these results in another cohort, OS and PFS were analyzed using bulk RNA sequencing data for patients with esophageal cancer obtained from TCGA. PFS was significantly better in the TLS^high^ group than in the TLS^low^ group (TLS^high^: 24.0 months; TLS^low^: 12.4 months; *p* = 0.049), and a similar trend was observed for OS (*p* = 0.103) (Supplementary Figure S1). A histological subgroup analysis showed no significant associations in any group; however, there was a trend toward improved OS and RFS values in the ESCC group. We identified a significant association between TLSs and survival in the ESCC histologic subtype, but not in EAC.

### 3.2. High TLS Density Is Associated with Superior Nutritional Status

**Figure 2:**
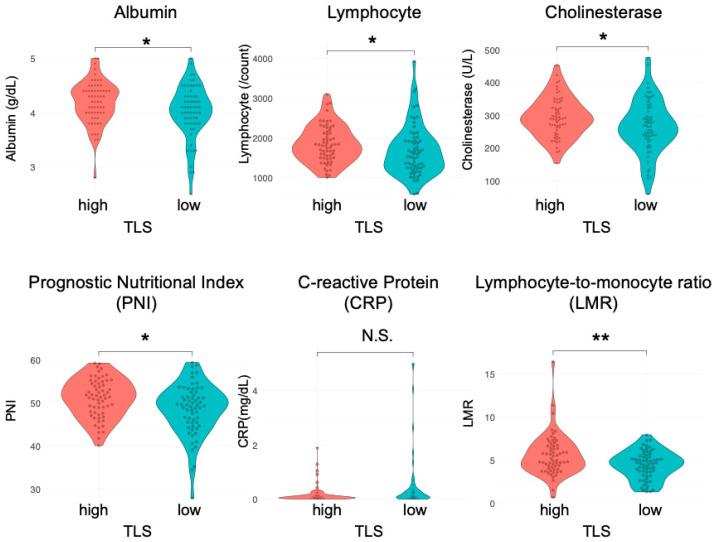
Correlation between preoperative blood test results and TLSs in patients with esophageal cancer. Comparison of the nutritional status (albumin level, lymphocyte count, cholinesterase, PNI, CRP, and LMR) of the TLS^high^ and TLS^low^ groups (*n* = 124, Mann–Whitney U test; *, *p* < 0.05; **, *p* < 0.01; N.S., not significant).

Considering the crucial role of nutritional status in immune responses [29,30], we examined the role of nutrition in TLS formation in esophageal cancer. Preoperative peripheral blood tests were retrospectively assessed in the same cohort. We assessed the LMR and PNI along with traditional nutritional indicators, such as albumin and cholinesterase. The LMR, which is determined using serum lymphocyte and monocyte counts, has been established as a prognostic factor in Hodgkin’s lymphoma and various gastrointestinal tumors [22,23]. The PNI, calculated from serum albumin levels and peripheral blood lymphocyte counts, is employed to evaluate the perioperative state and predict outcomes in patients with malignant tumors of the gastrointestinal tract [24]. The median serum albumin levels, peripheral blood lymphocyte counts, cholinesterase levels, PNI, and LMR were significantly higher in the TLS^high^ group than in the TLS^low^ group (Figure 2; albumin (g/dL): 4.2 IQR: 4.0–4.4) vs. 4.1 (IQR: 3.8–4.3), *p* = 0.048; lymphocyte (count): 1811 (IQR: 1497–2146) vs. 1596 (IQR: 1243–1922), *p* = 0.031; cholinesterase (U/L): 290 (IQR: 256–336) vs. 265 (IQR: 225–319), *p* = 0.033; PNI: 51.5 (IQR: 47.7–54.6) vs. 49.5 (IQR: 45.5–52.4), *p* = 0.012; LMR: 5.09 (IQR: 4.09–6.67) vs. 4.62 (IQR:3.37–5.49), *p* = 0.009, respectively). The median C-reactive protein (CRP) levels (mg/dL) showed a similar trend (0.09 vs. 0.13, *p* = 0.056, respectively). However, the neutrophil, monocyte, and platelet levels and the platelet-to-lymphocyte and neutrophil-to-lymphocyte ratios did not differ between the two groups (Supplementary Figure S2). In subgroup analyses, albumin, lymphocytes, cholinesterase, PNI, and LMR were significantly correlated with TLSs in the ESCC group, while no correlations were detected in the EAC group (Supplementary Figure S3).

### 3.3. TLSs Affect the Macrophage Phenotype

**Figure 3:**
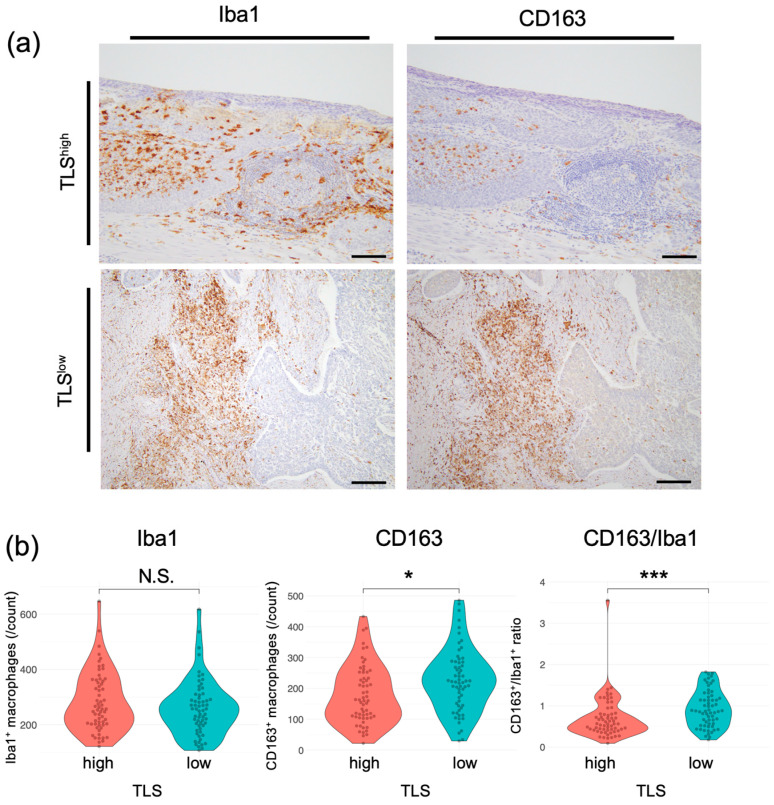
Correlation between TLSs and macrophages in patients with esophageal cancer. (**a**) Representative images of specimens that were subjected to IHC. Left, whole macrophages; right, M2 macrophages. Scale bars: 100 µm. (**b**) Comparison of macrophages in the TLS^high^ and TLS^low^ groups. Left, whole macrophages; center, M2 macrophages; right, CD163^+^/Iba1^+^ cell ratio (*n* = 124, Mann–Whitney U test; *, *p* < 0.05; ***, *p* < 0.001, N.S., not significant).

We next investigated whether there were differences in individual cell types, including T-cell subsets, macrophages, and plasma cells, using esophageal cancer specimens. No significant differences in the CD8+ T-cell or regulatory T-cell (Treg) count were observed between TLS^high^ and TLS^low^ tumors in intratumoral tissues in this cohort, including in subgroup analyses (Supplementary Figure S4). The counts of Iba1^+^ cells, a pan-macrophage marker, did not differ significantly between the two groups (Figure 3a,b; median, TLS^high^: 257, TLS^low^: 255; *p* = 0.297). In contrast, the counts of CD163^+^ cells, indicative of M2 macrophages, which promote tumorigenesis and development [31], were significantly lower in the TLS^high^ group than in the TLS^low^ group (median, TLS^high^: 162; TLS^low^: 222; *p* = 0.013). The proportion of M2 macrophages among the total macrophages was also lower in the TLS^high^ group than in the TLS^low^ group. The ESCC group also showed significant differences in CD163 and CD163/Iba1 (Supplementary Figure S5). No significant correlations were detected in the EAC group. These results suggest that a low TLS score is correlated with a high number of M2 macrophages, resulting in an immunosuppressive environment. Conversely, elevated TLS levels are correlated with an increase in M1 macrophages, which can kill and remove tumor cells [32].

### 3.4. MUM-1^+^ Plasma Cells Accumulate in TLSs to Activate Tumor Immunity

**Figure 4:**
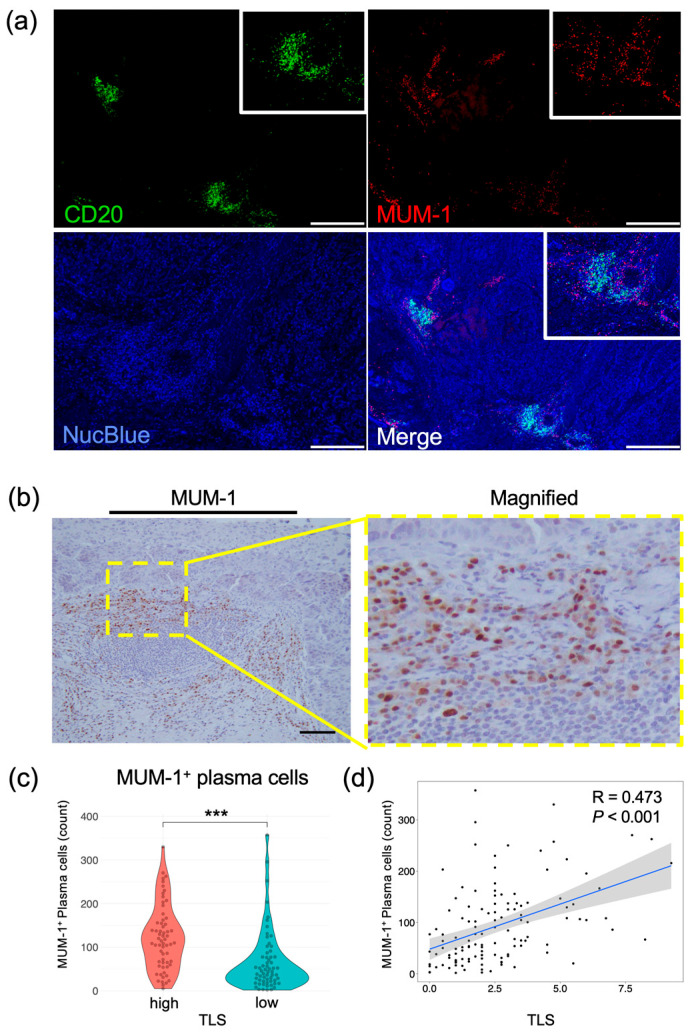
Evaluation of plasma cells in esophageal cancer specimens. (**a**) Representative images of multiplex immunofluorescent images. Specimens stained with CD20 (green), MUM-1 (red), and NucBlue (blue) are shown. Insets show enlarged images. Scale bars: 500 µm. (**b**) Representative IHC images showing MUM-1 expression. The yellow rectangle shows the location of the magnified image. Scale bar: 100 µm. (**c**) Comparison of plasma cells in the TLS^high^ and TLS^low^ groups (*n* = 124, Mann–Whitney U test; ***, *p* < 0.001). (**d**) Scatterplot of TLSs and MUM-1 expression levels. Spearman’s correlation coefficient was calculated. The black dots represent individual data points. The blue line indicates the regression line, and the shaded area shows the 95% confidence interval.

To explore the relationship between B cells and plasma cells that produce IgG or IgA antibodies targeting tumor-associated antigens [8], we analyzed the positional relationship between CD20^+^ B cells and MUM-1^+^ plasma cells using multiplex IF with resected esophageal tumor specimens (Figure 4a). Figure 4a shows that MUM-1^+^ plasma cells are accumulated near clusters of CD20^+^ B cells. To prove the correlation between MUM-1^+^ plasma cells and B-cell clusters in esophageal cancers, MUM-1^+^ cells were counted and evaluated using IHC in the same cohort (Figure 4b). The number of MUM-1^+^ cells was significantly higher in the TLS^high^ group than in the TLS^low^ group (Figure 4c), and the numbers of TLSs and MUM-1^+^ plasma cells were strongly correlated in the overall analysis and in the ESCC subgroup (Figure 4d and Supplementary Figure S6). These results demonstrate that an increase in TLSs is closely associated with a high degree of infiltration of MUM-1^+^ plasma cells.

### 3.5. FAP^+^ CAFs Have a Negative Correlation with TLS Formation

**Figure 5:**
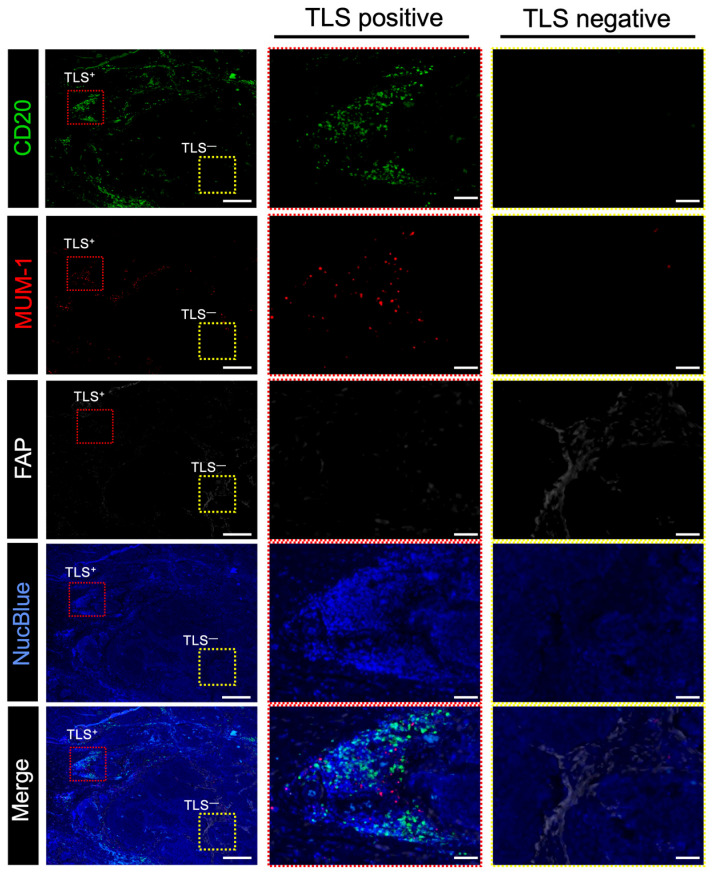
Relationship between FAP^+^ CAFs and TLSs in surgically resected human esophageal cancer samples. Representative multiplex immunofluorescence-stained images. Specimens stained with CD20 (green), MUM-1 (red), FAP (white), and NucBlue (blue) are shown. (**Left**), representative images of the esophageal cancer specimens; center, magnified images showing the location of TLS-positive cells; (**right**), magnified images showing the location of TLS-negative cells. Red and yellow rectangles indicate the locations with high and low TLS counts in the magnified images, respectively. Scale bar: 500 μm; scale bar in magnified images: 100 μm.

**Figure 6:**
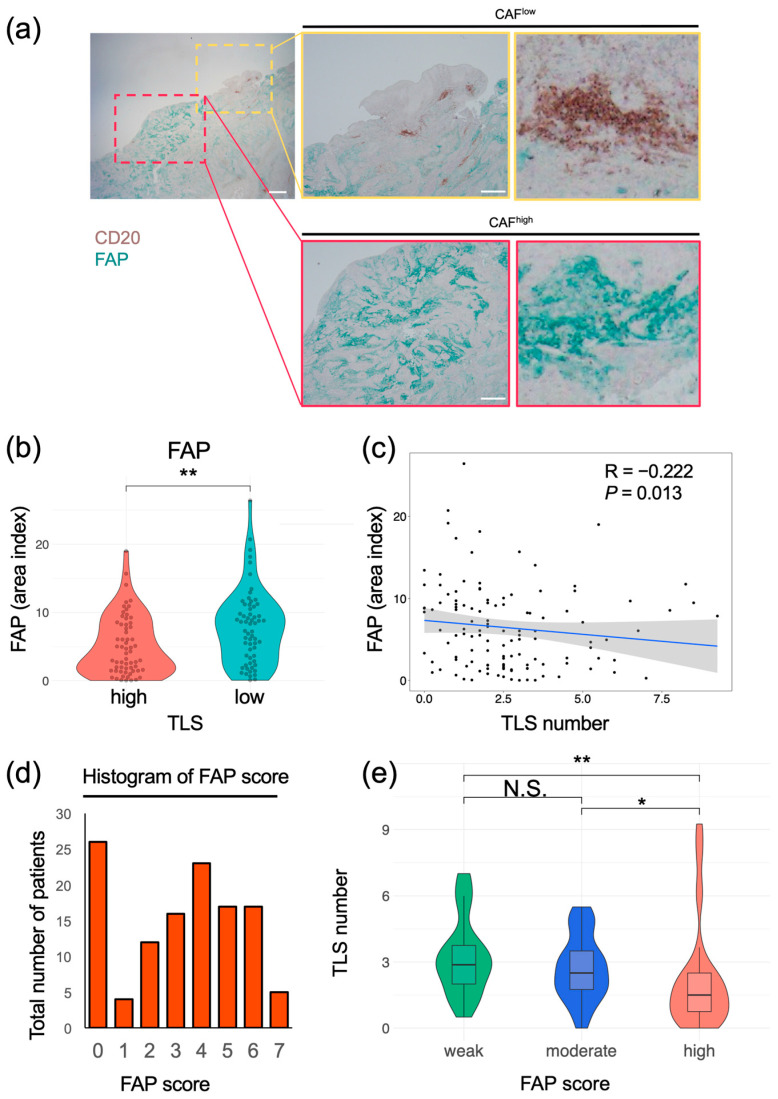
Correlation between FAP^+^ CAFs and TLS in esophageal cancer tissues. (**a**) Representative images of samples subjected to double staining and IHC. DAB, CD20; cyan, FAP. Yellow rectangles show sites with a high number of TLSs, and red rectangles show sites with a low number of TLSs. Scale bars: 1000 μm (left) and 500 μm (middle) (**b**) Comparison of FAP area indices of the TLS^high^ and TLS^low^ groups (Mann–Whitney U test; **, *p* < 0.01). (**c**) Distribution of TLS numbers according to FAP area index. Spearman’s correlation coefficient was calculated. The black dots represent individual data points. The blue line indicates the regression line, and the shaded area shows the 95% confidence interval. (**d**) Distribution of patients according to FAP scores. (**e**) TLS numbers were compared with FAP scores in three groups (≥5, high; 2–4, moderate; and 0 or 1, weak) (Kruskal–Wallis test; *, *p* < 0.05; **, *p* < 0.01; N.S., not significant). ((**a**–**e**); *n* = 124).

To verify the interaction between TLSs and CAFs, their localization in esophageal cancer specimens was determined using multiplex IF. In the same specimen, areas containing TLSs, composed of CD20^+^ B cells and MUM-1^+^ plasma cells, had a minimal number of FAP^+^ cells. Conversely, the areas with abundant FAP^+^ cells showed no detectable TLS formation (Figure 5). To quantify the correlation between CAFs and TLSs, we evaluated TLS clusters with CD20^+^ B cells and FAP^+^ cells using multiplex IHC in the same cohort. Similarly to the results obtained using IF, TLS clusters containing CD20^+^ and FAP^+^ cells could be distinguished using IHC (Figure 6a). The area index of FAP^+^ cells was significantly smaller in the TLS^high^ group than in the TLS^low^ group (Figure 6b; median: TLS^high^, 4.14; TLS^low^, 7.82; *p* = 0.003). The number of TLSs and the FAP area index were significantly negatively correlated (Figure 6c; R = −0.22; *p* = 0.013). In a subgroup analysis, a significant inverse correlation between TLSs and the FAP area index was detected in the ESCC group (Supplementary Figure S7). We re-evaluated the correlation using modified FAP scores that were previously proposed at our institute and calculated based on intensity and proportion [19]. TLS numbers decreased significantly in a FAP score-dependent manner (Figure 6d,e). Using αSMA, another typical marker associated with CAFs, similar results were obtained (Supplementary Figure S8). These results suggest that FAP^+^ CAFs negatively affect TLS formation.

## 4. Discussion

There are two main types of esophageal cancer: ESCC and EAC. In our cohort, TLSs in the ESCC subgroup were strongly associated with the prognosis, nutritional status, and the number of plasma cells, while no correlation was detected in EAC. The reason for this difference between types is not clear and cannot be conclusively stated; however, since SCC has a higher tumor mutation burden and is more immunogenic [33], it is possible that tumor immunity is more likely to be activated in SCC via TLSs, leading to a better response to immunotherapy. Postoperative nivolumab therapy in patients with esophageal cancer who do not achieve a pathologic complete response to preoperative chemoradiotherapy is more beneficial in squamous cell carcinoma than in adenocarcinoma [4]. TLSs may play a role in the difference in response between histologic types (i.e., the stronger associations between TLSs and outcomes, FAP^+^ CAFs, and plasma cells observed in ESCC compared to EAC). Given the limited number of EAC cases in Japan, it is difficult to reach definitive conclusions in a single institution. A worldwide multicenter study is needed to evaluate histological types to assess the generalizability of the results.

A high level of TLS formation is associated with a better prognosis in ESCC. In an analysis of publicly available TCGA RNA sequencing data, high TLS-related gene expression was also related to longer PFS in ESCC prognosis; however, it did not have a significant impact on OS, differing from the results for our cohort. This discrepancy may be attributed to the fact that in the publicly available data, patients were grouped based on bulk RNA expression levels. Because the degree of TLS clustering within the tumor appears to be immunologically critical, rather than the overall level of TLS-related RNA expression, IHC evaluation of resected tumor specimens may provide more meaningful insights into tumor-associated TLSs than bulk RNA-based analyses. However, RNA evaluations of TLSs may reveal candidate prognostic biomarkers.

In this study, a significant correlation between TLSs and MUM-1^+^ plasma cells in intratumoral tissues was detected. This suggests that TLSs serve as a local site of B-cell maturation, resulting in the proliferation of MUM-1^+^ plasma cells within the tumor [10]. Additionally, tumors with a high number of TLSs have tumor-specific antibodies, leading to increased antibody-dependent cellular cytotoxicity (ADCC), antibody-dependent cell phagocytosis (ADCP) [34], and complement-dependent cytotoxicity (CDC), consistent with the high number of M1 macrophages in the TLS^high^ group. These mechanisms are consistent with previous observations, including the fact that the activation of humoral immunity can produce an antitumor effect [9,35,36]. Thus, TLSs have the potential to serve as biomarkers of tumor immunity and possess prognostic significance in the management of esophageal cancer.

Many factors have been reported to increase the number of TLSs or activate TLS formation via TLS-associated cytokines and chemokines, including lymphotoxin [37], tumor necrosis factor alpha (TNFα) [38,39], LIGHT (TNFS14) [40], CCL21, CCL19, and CXCL12 [41]; however, none of these factors have been applied in clinical settings. This study revealed a significant positive association between the number of TLSs and nutritional status (Figure 2), suggesting that improving host nutrition may activate TLS formation and other tumor-immunity-related mechanisms. Peripheral blood tests that correlate with TLS formation have the potential to predict the status of host tumor immunity and prognosis.

FAP^+^ CAFs were negatively associated with intratumoral TLSs, suggesting that tumor immunosuppression occurs via the inhibition of TLS cluster formation and plasma cell maturation (Figure 5 and Figure 6). Although assessing the CAF status is beneficial in tumor immunology, a highly specific marker for evaluating CAFs has yet to be defined, since CAFs are derived from several different cell types [42]. Recently, a protocol using two markers, podoplanin (PDPN) and FAP, has been proposed for CAF identification; PDPN^+^ FAP^−^ CAFs, as lymphoid tissue organizer phenotypes, identified the population that promotes and organizes tumor-associated TLS formation [21]. This classification is currently the most effective method for analyzing TLSs and tumor immunity to date. However, the approach is complex, requiring multiple staining steps and classification if the number of clinical specimens is large. To resolve this problem, we adopted a simplified approach involving a single-stain IHC evaluation of FAP^+^ CAFs, which are widely recognized as a tumor-promoting and immunosuppressive subtype of CAFs closely associated with tumor progression and poor clinical outcomes [19,20,43,44,45]. As expected, a significant negative association between FAP^+^ CAFs and TLSs was detected in ESCC, supporting the clinical value of single staining for FAP as an alternative to multiple staining for FAP and PDPN.

Another advantage of using FAP as a CAF marker is its therapeutic applications. Selective FAP^+^ CAF deletion may contribute to an increase in TLSs because the numbers of FAP^+^ CAFs and TLSs are negatively correlated. Recently, several therapeutic approaches have been developed to target FAP^+^ CAFs, aiming to overcome tumor immunosuppression. Experimental strategies include adenoviral-vector vaccines designed to eliminate FAP^+^ cells [46], small-molecule FAP inhibitors such as talabostat [47], and CAR-T cell therapies targeting FAP [48], all of which have demonstrated antitumor efficacy in preclinical models. Furthermore, we have previously developed near-infrared photoimmunotherapy (NIR-PIT) to target FAP^+^ CAFs utilizing sibrotuzumab, which is an anti-human FAP antibody and was clinically used in a phase I/II study [49,50]. This can selectively kill only CAFs at the tumor site after irradiation with NIR light [51]. As NIR-PIT is already clinically used for patients with advanced head and neck cancers in Japan, this technology is expected to be incorporated into clinical practice for the treatment of esophageal cancer. For these reasons, employing FAP as a CAF marker is advantageous for both diagnostic and therapeutic purposes.

This study has some limitations. First, we evaluated clinical specimens from patients with esophageal cancer at a single institution, and the number of cases—particularly those with adenocarcinoma—was limited. Therefore, further studies with larger multicenter cohorts are warranted to validate our findings and improve their generalizability. Second, although there was a significant negative association between TLSs and CAFs, we did not determine the mechanism by which FAP^+^ CAFs suppress TLS formation or the role of TLSs in tumor suppression via humoral-immunity-related mechanisms. Further investigations are needed to understand the efficacy of TLSs and their potential prognostic value.

## 5. Conclusions

We demonstrated that TLSs are an independent prognostic factor in esophageal cancer. This may be attributed to their effects on the TME, influencing both plasma cells and M1 macrophages. Moreover, FAP^+^ CAFs were negatively correlated with the number of TLSs. Overall, TLSs are potential biomarkers and prognostic factors for patients with esophageal cancer, and CAF-targeted therapy could be a useful strategy to improve host tumor immunity.

## Data Availability

The datasets generated and/or analyzed in the current study are available from the corresponding author upon reasonable request.

## Supplementary Materials

The following supporting information can be downloaded at: https://www.mdpi.com/article/10.3390/cancers17203351/s1, Supplementary Figure S1. Survival analyses by OS and PFS in TCGA cohort data; Supplementary Figure S2. Comparison of TLS with hematological and biochemical test indices; Supplementary Figure S3. Correlation between preoperative blood test results and TLSs in patients with esophageal cancer in histological related subgroup analysis; Supplementary Figure S4. Relationship between CD8+, FoxP3+ tumor-infiltrating lymphocytes and TLS in resected esophageal cancer samples; Supplementary Figure S5. Correlation between TLSs and macrophages in patients with esophageal cancer in histological related subgroup analysis; Supplementary Figure S6. Evaluation of plasma cells in esophageal cancer specimens in ESCC; Supplementary Figure S7. Correlation between FAP+ CAFs and TLS in ESCC tissues; Supplementary Figure S8. Correlation of αSMA+ CAFs and TLS distribution in esophageal cancer tissues; Supplementary Table S1. The correlation between TLS groups and clinicopathological characteristics of esophageal cancer; Supplementary Table S2. multivariate analyses of prognostic factors associated with overall survival and progression-free survival in all esophageal cancer patients and in those with ESCC.
