# Supplementary material for: Tertiary Lymphoid Structures Are Associated with Favorable Clinical Outcomes and Negatively Correlated with Cancer-Associated Fibroblasts in Esophageal Cancer
Source: Cancers (Basel). 2025 Oct 17;17(20):3351. doi: 10.3390/cancers17203351 (PMC12562458; doi:10.3390/cancers17203351)
Supplement: Supplementary file 1 [file cancers-17-03351-s001.zip › cancers-3892246-supplementary.pdf]

Supplementary Information for

Tertiary Lymphoid Structures Are Associated with Favorable Clinical Outcomes and Negatively Correlated with Cancer-Associated Fibroblasts in Esophageal Cancer

Authors and affiliations:

Tomoyoshi Kunitomo[1], Kazuhiro Noma[1], Noriyuki Nishiwaki[1], Seitaro Nishimura[1], Yasushige Takeda[1], Hijiri Matsumoto[1], Tatsuya Takahashi[1], Kento Kawasaki[1], Masaaki Akai[1], Naoaki Maeda[1], Shunsuke Tanabe[1], Toshiaki Ohara[1,2], Hiroshi Tazawa[1,3], Yasuhiro Shirakawa[1,4], and Toshiyoshi Fujiwara[1]

[1] Department of Gastroenterological Surgery, Graduate School of Medicine, Dentistry and Pharmaceutical Sciences, Okayama University, Okayama, Japan.

[2] Department of Pathology & Experimental Medicine, Graduate School of Medicine, Dentistry and Pharmaceutical Sciences, Okayama University, Okayama, Japan.

[3] Center for Innovative Clinical Medicine, Okayama University Hospital, Okayama, Japan.

[4] Department of Surgery, Hiroshima City Hiroshima Citizens Hospital, Hiroshima, Japan.

Corresponding author:

Kazuhiro Noma, MD, PhD
Associate Professor, Senior Lecturer
Department of Gastroenterological Surgery, Okayama University Graduate School of Medicine, Dentistry and Pharmaceutical Sciences, Okayama, Japan.
2-5-1 Shikata-cho, Kita-ku, Okayama 700-8558, Japan
Tel.: +81-(86)-235-7257, +81-86-221-8775
Email: knoma@md.okayama-u.ac.jp

List of Supplementary Information

Supplementary Figure S1. Survival analyses by OS and PFS in TCGA cohort data.

Supplementary Figure S2. Comparison of TLS with hematological and biochemical test indices.

Supplementary Figure S3. Correlation between preoperative blood test results and TLSs in patients with esophageal cancer in histological related subgroup analysis.

Supplementary Figure S4. Relationship between $CD8^+$, $FoxP3^+$ tumor-infiltrating lymphocytes and TLS in resected esophageal cancer samples.

Supplementary Figure S5. Correlation between TLSs and macrophages in patients with esophageal cancer in histological related subgroup analysis

Supplementary Figure S6. Evaluation of plasma cells in esophageal cancer specimens in ESCC

Supplementary Figure S7. Correlation between FAP^+ CAFs and TLS in ESCC tissues.

Supplementary Figure S8. Correlation of αSMA^+ CAFs and TLS distribution in esophageal cancer tissues.

Supplementary Table S1. The correlation between TLS groups and clinicopathological characteristics of esophageal cancer

Supplementary Table S2. multivariate analyses of prognostic factors associated with overall survival and progression-free survival in all esophageal cancer patients and in those with ESCC

Supplementary Figure S1

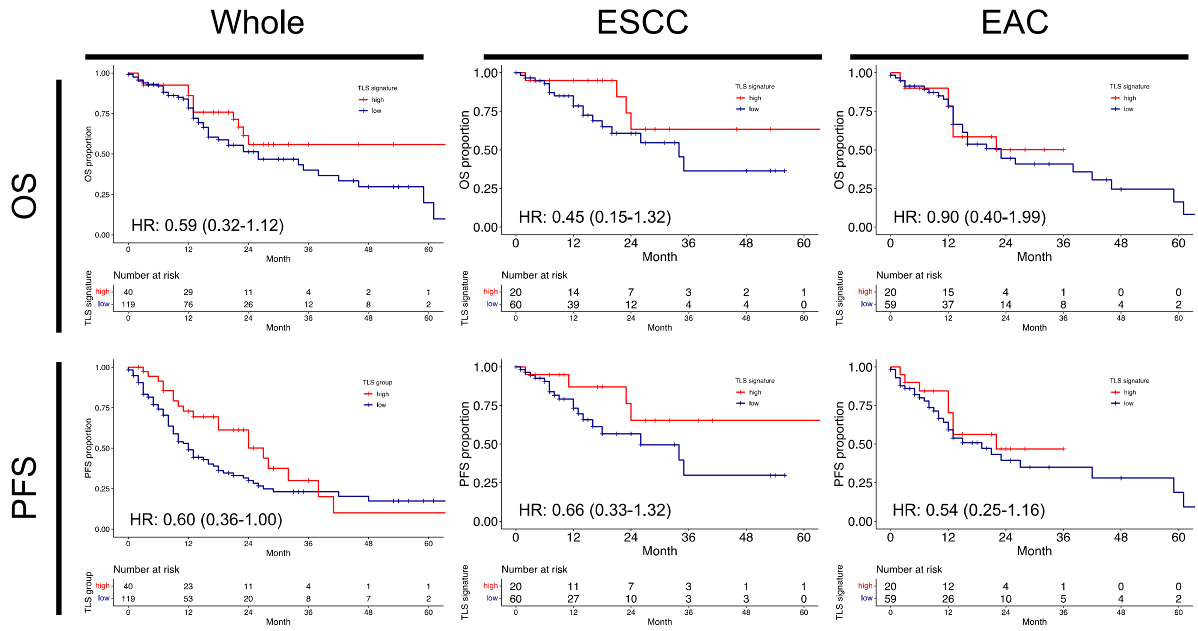

Supplementary Figure S1. Survival analyses by OS and PFS in TCGA cohort data

Survival outcomes in TCGA cohort data according to TLS$^{high/low}$ groups (n = 159) and by histological subgroup, SCC (n = 80) or adenocarcinoma (n = 79) were shown (Cox regression hazard model; HR, hazard ratio with 95% confidence intervals).

Supplementary Figure S2

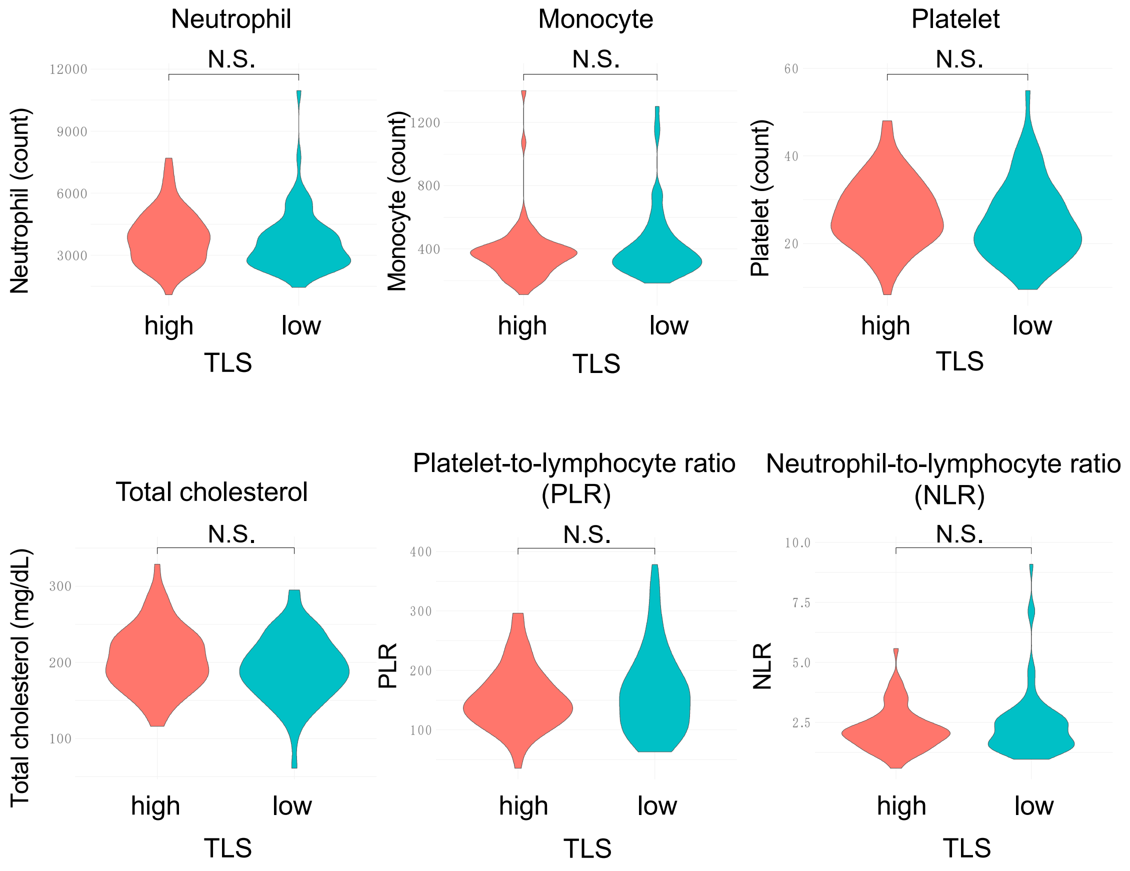

Supplementary Figure S2. Comparison of TLS with hematological and biochemical test indices

Comparison of hematological and biochemical status between TLS$^{high/low}$ groups. (n = 124, Mann–Whitney U test; PLR, platelet-to-lymphocyte ratio; NLR, neutrophil-to-lymphocyte ratio; N.S., not significant).

Supplementary Figure S3

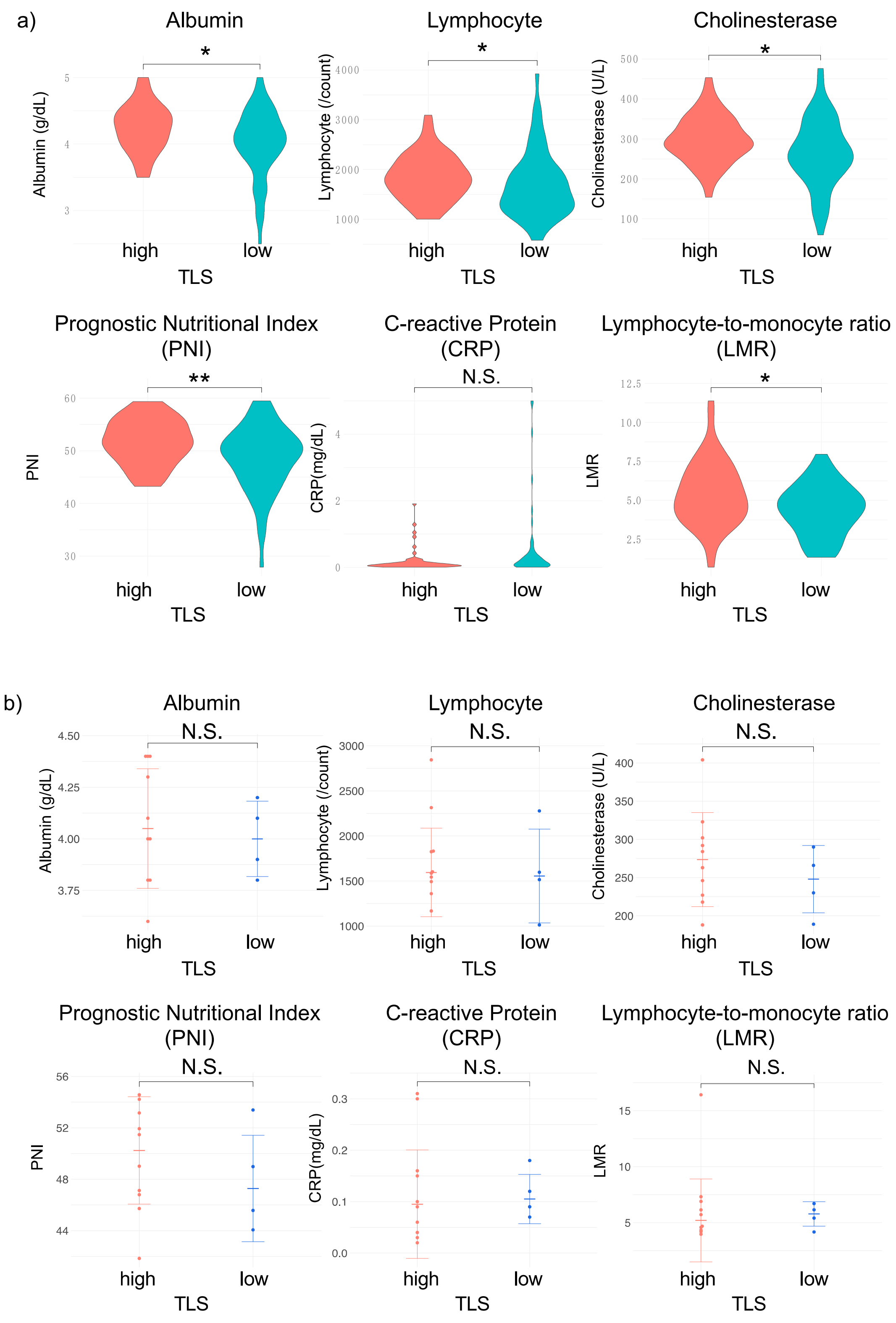

Supplementary Figure S3. Correlation between preoperative blood test results and TLSs in patients with esophageal cancer in histological related subgroup analysis

Comparison of the nutritional status (albumin level, lymphocyte count, cholinesterase, PNI, CRP, and LMR) of the TLS^{high} and TLS^{low} groups (a) in ESCC group ($n = 106$) and (b) in EAC group ($n = 14$) (Mann–Whitney U test; *$P < 0.05$; **$P < 0.01$).

Supplementary Figure S4

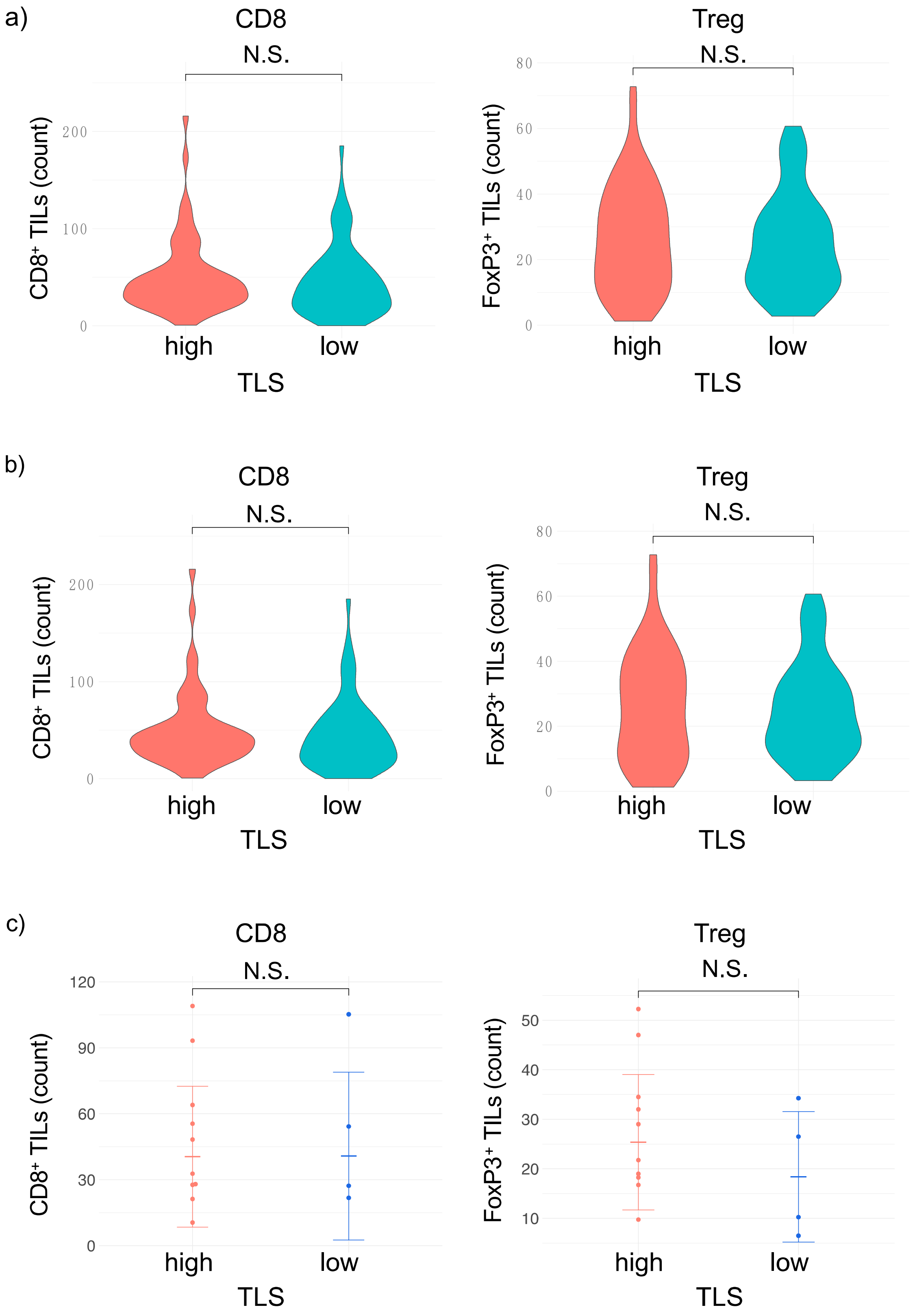

Supplementary Figure S4. Relationship between $CD8^+$, $FoxP3^+$ tumor-infiltrating lymphocytes and TLS in resected esophageal cancer samples

Comparison of TILs in intra-tumoral tissues in esophageal cancer between $TLS^{high/low}$ groups (a) in all cases (*n* = 124), (b) in ESCC (*n* = 106), and (c) EAC (*n* = 14). Left, $CD8^+$ TILs; right, $FoxP3^+$ TILs. Mann-Whitney U test; N.S., not significant.

Supplementary Figure S5

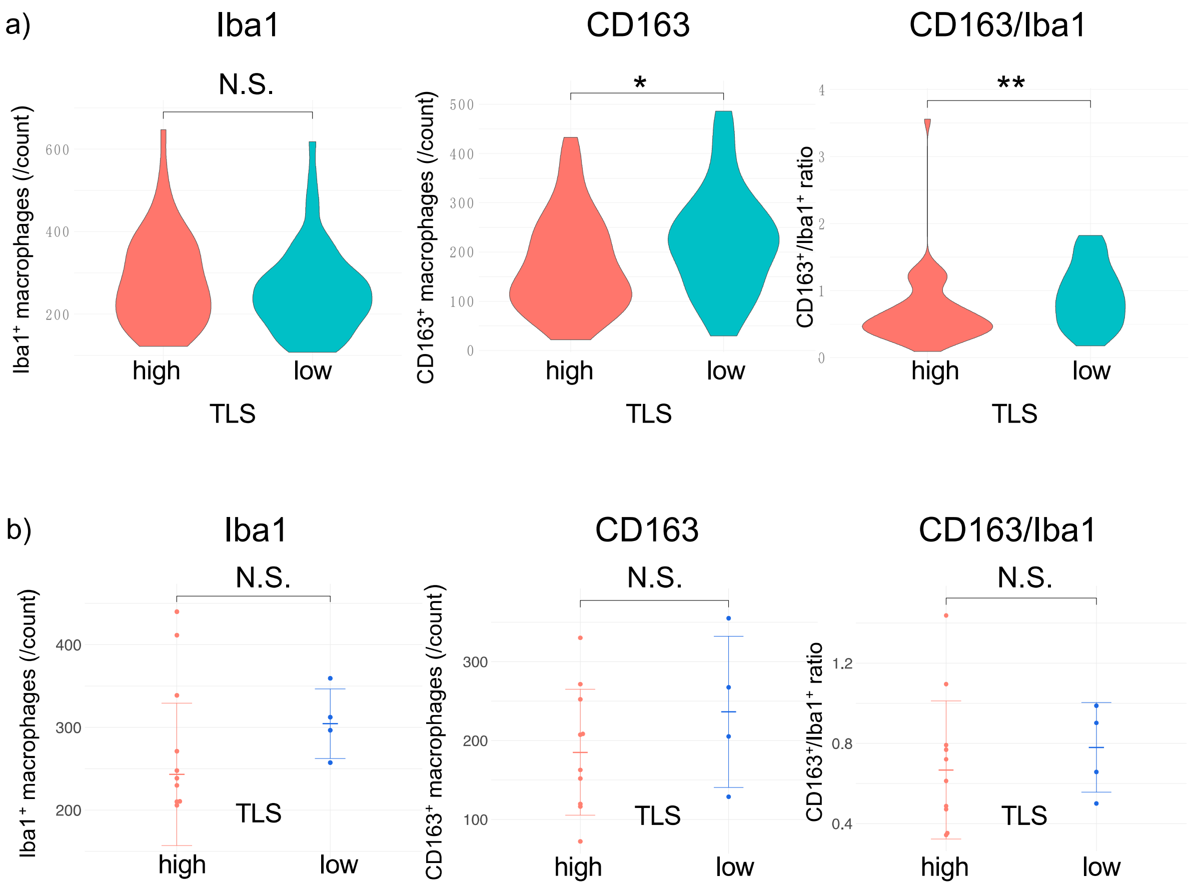

Supplementary Figure S5. Correlation between TLSs and macrophages in patients with esophageal cancer in histological related subgroup analysis

Comparison of macrophages in the TLS^{high} and TLS^{low} groups (a) in ESCC group (n = 106) and (b) in EAC group (n = 14). Left, whole macrophages; center, M2 macrophages; right, CD163^{+}/Iba1^{+} cell ratio (Mann–Whitney U test; *$P < 0.05$; **$P < 0.01$).

Supplementary Figure S6

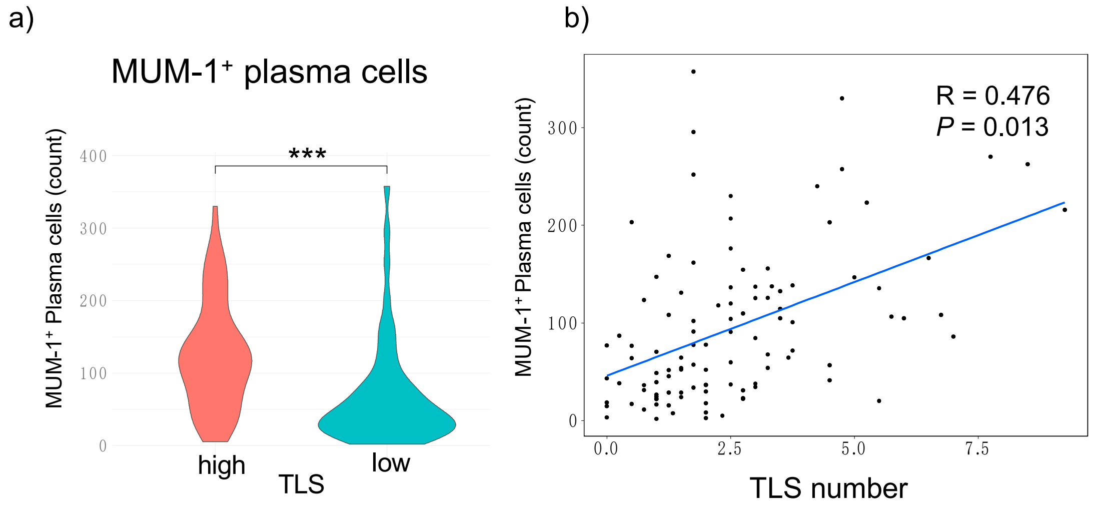

Supplementary Figure S6. Evaluation of plasma cells in esophageal cancer specimens in ESCC

(a) Comparison of plasma cells in the TLS^{high} and TLS^{low} groups in ESCC (n = 106, Mann–Whitney U test; ***$P < 0.001$). (b) Scatterplot of TLSs and MUM-1 expression levels in ESCC. Spearman's correlation coefficient was calculated. The black dots represent individual data points. The blue line indicates the regression line, and the shaded area shows the 95% confidence interval.

Supplementary Figure S7

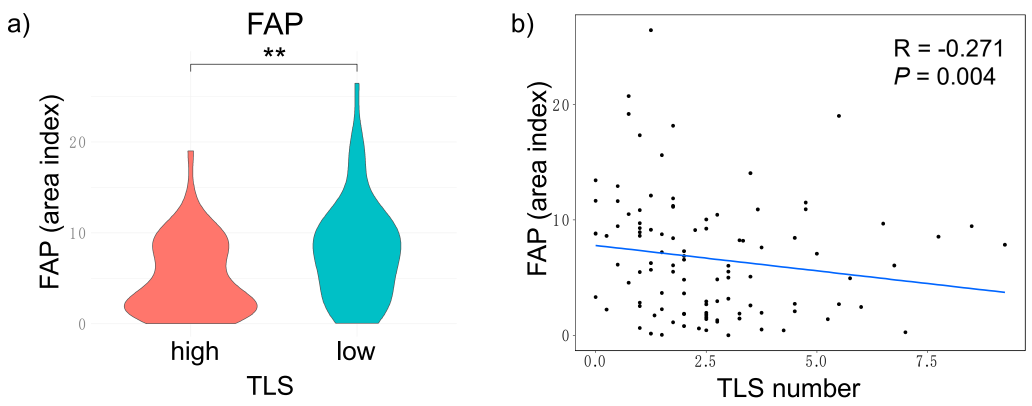

Supplementary Figure S7. Correlation between FAP^{+} CAFs and TLS in ESCC tissues. (a) Comparison of FAP area indices of the TLS^{high} and TLS^{low} groups in ESCC samples. (Mann–Whitney U test; $**P < 0.01$). (b) The correlation between TLSs and FAP area indices is shown using a scatter plot. Spearman's correlation coefficient was calculated. The black dots represent individual data points. The blue line indicates the regression line, and the shaded area shows the 95% confidence interval.

Supplementary Figure S8

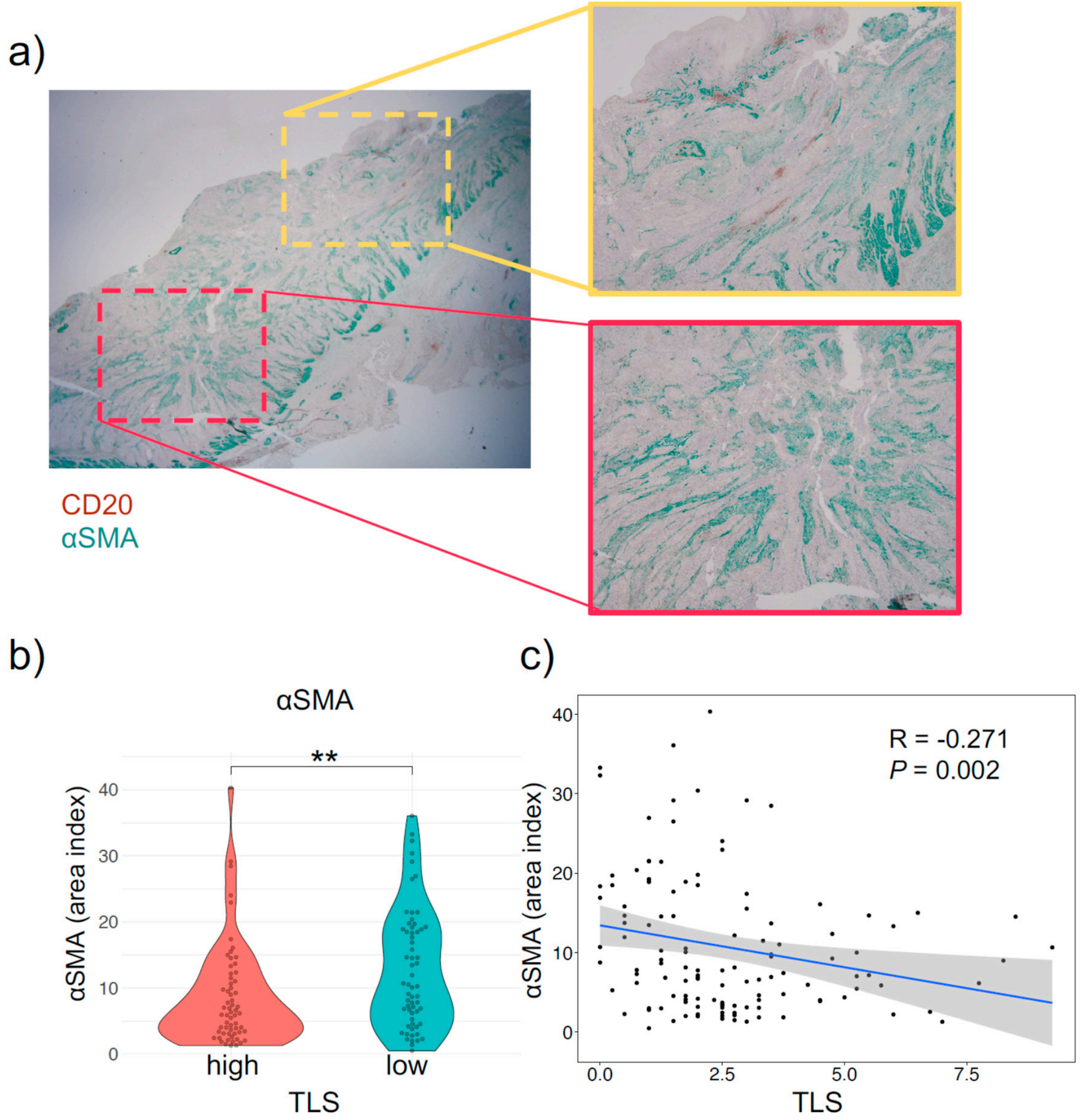

Supplementary Figure S8. Correlation of αSMA^{+} CAFs and TLS distribution in esophageal cancer tissues.

(a) Representative images of double staining IHC images. DAB, CD20; cyan, αSMA. Yellow rectangle shows the location of high TLS, and red rectangle shows the location of low TLS.

(b) Comparison of αSMA area index between TLS$^{high/low}$ groups. (Mann–Whitney U test; **, $P < 0.01$). (c) The correlation between TLS and αSMA area index is shown by scatter plot.

Spearman's correlation coefficient. The black dots represent individual data points. The blue line indicates the regression line, and the shaded area shows the 95% confidence interval.

Supplementary Table S1. The correlation between TLS groups and clinicopathological characteristics of esophageal cancer

	TLS group		
	Low	High	p value
Factors	*n* =62	*n* =62	
Age (Year), median（IQR)	66.0 (60-71.0)	67.5 (63.3-73)	0.102§
Gender			
Male	57 (53.8%)	49 (46.2%)	0.072†
Female	5 (27.8%)	13 (72.2%)	
Hight (cm), Median (IQR)	165 (160-169)	162 (157-169)	0.404§
Weight (kg), Median (IQR)	58.0(51.9-62.6)	57.1 (52.8-65.9)	0.624§
Body Mass Index (kg/m^2), Median (IQR)	21.7 (19.8-23.2)	22.4 (20.4-24.2)	0.158§
Neoadjuvant chemotherapy			
None	49 (79.0%)	56 (90.3%)	0.133†
Yes	13 (21.0%)	6 (9.7%)	
Histological type			0.213†
Squamous cell carcinoma	55 (51.9%)	51 (48.1%)	
Adenocarcinoma	4 (28.6%)	10 (71.5%)	
Others	3 (75.0%)	1 (25.0%)	
Location			0.144†
Ce	6 (66.7%)	3 (33.3%)	
Ut	9 (40.9%)	13 (59.1%)	
Mt	32 (56.1%)	25 (43.9%)	
Lt	12 (57.1%)	9 (42.9%)	
Ae	2 (20.0%)	8 (80.0%)	
EGJ	1 (20.0%)	4 (80.0%)	
pT			0.062†
Tis	1 (50.0%)	1 (50.0%)	
T1	20 (36.4%)	35 (63.6%)	
T2	7 (58.3%)	5 (41.7%)	
T3	32 (62.7%)	19 (37.3%)	

T4a	2 (50.0%)	2 (50.0%)	
pN			0.307†
N0	24 (41.4%)	34 (58.6%)	
N1	17 (56.7%)	13 (43.3%)	
N2	16 (61.5%)	10 (38.5%)	
N3	5 (50.0))	5 (50.0%)	
pM			1†
M1	4 (6.5%)	4 (6.5%)	
pStage			0.256†
0	1 (50.0%)	1 (50.0%)	
I	15 (35.7%)	27 (64.3%)	
II	13 (54.2%)	11 (45.8.%)	
III	22 (57.9%)	16 (42.1%)	
IVA	7 (70.0%)	3 (30.0%)	
IVB	4 (50.0%)	4 (50.0%)	

§, Mann–Whitney *U* test; †, Fisher's exact test; IQR, Interquartile range

Supplementary Table S2. multivariate analyses of prognostic factors associated with overall survival and progression-free survival in all esophageal cancer patients and in those with ESCC

	Overall survival					
	Univariate			Multivariate		
	HR	95%CI	p-value	HR	95%CI	p-value
Age						
≧65	1.73	1.00-3.00	0.05			
Gender						
Male	3.71	1.16-11.84	0.027*	2.33	0.71-7.6	0.161
BMI						
<18.5	1.79	0.97-3.30	0.062			
Neoadjuvant chemotherapy						
Yes	2.44	1.36-4.40	0.003*			
Tumor location						
Ce, Ut	1.44	0.82-2.51	0.205			
Histological type						
SCC	1.58	0.72-3.46	0.257			
Tumor depth						
pT3,4	3.86	2.30-6.49	<0.001*	2.24	1.25-4.04	0.007*
Lymph node metastasis						
N (+)	3.51	2.02-6.09	<0.001*	2.26	1.20-4.24	0.011*
Distant organ metastasis						
M (+)	2.36	1.01-5.50	0.047*			
TLS number						
high	0.32	0.19-0.55	<0.001*	0.36	0.21-0.63	<0.001*
CD8+T cell						
high	0.69	0.42-1.14	0.145	0.61	0.36-1.02	0.058
Treg (FoxP3+)						
high	2.82	1.67-4.76	<0.001*	1.99	1.15-3.44	0.014*
TAM (CD163+)						
high	1.84	1.12-3.04	0.017*			

FAP			
high	2.79	1.63-4.76	<0.001*

Cox's proportional hazards model, AIC in a Stepwise Algorithm, statistical significance: P-value *<0.05, HR: Hazard ratio, CI: confidence interval, BMI: Body Mass Index, SCC: squamous cell carcinoma

	Progression-free survival					
	Univariate			Multivariate		
	HR	95%CI	p-value	HR	95%CI	p-value
Age						
≧65	1.36	0.81-2.29	0.247			
Gender						
Male	4.26	1.33-13.61	0.014*	3.06	0.94-9.99	0.063
BMI						
<18.5	1.59	0.86-2.91	0.138			
Neoadjuvant chemotherapy						
Yes	3.54	1.99-6.28	<0.001*	1.97	1.04-3.71	0.037*
Tumorlocation						
Ce,Ut	1.23	0.71-2.15	0.465			
Histologicaltype						
SCC	1.75	0.80-3.82	0.164			
Tumor depth						
pT3,4	4.20	2.54-6.95	<0.001*	2.25	1.24-4.07	0.008*
Lymph node metastasis						
N(+)	3.44	2.03-5.84	<0.001*	1.90	1.02-3.54	0.043*
Distant organ metastasis						
M(+)	2.69	1.15-6.26	0.022*			
TLS number						
high	0.32	0.19-0.54	<0.001*	0.42	0.24-0.72	0.002*
CD8+T cell						
high	0.72	0.44-1.17	0.181	0.63	0.38-1.05	0.079
Treg (FoxP3+)						
high	3.13	1.86-5.26	<0.001*	2.26	1.29-3.99	0.005*

	HR	95%CI	p-value	HR	95%CI	p-value
TAM (CD163+)						
high	2.06	1.26-3.37	0.004*			
FAP						
high	2.90	1.73-4.85	<0.001*			

Cox‘s proportional hazards model, AIC in a Stepwise Algorithm, statistical significance: P-value *<0.05, HR: Hazard ratio, CI: confidence interval, BMI: Body Mass Index, SCC: squamous cell carcinoma

Subgroup analysis in ESCC

	Overall survival					
	Univariate			Multivariate		
	HR	95%CI	p-value	HR	95%CI	p-value
Age						
≧65	1.83	1.03-3.25	0.039*			
Gender						
Male	4.99	1.21-20.5	0.036*	3.33	0.78-14.2	0.10
BMI						
<18.5	2.34	1.23-4.45	0.010*	1.84	0.93-3.65	0.079
Neoadjuvant chemotherapy						
Yes	3.3	1.79-6.07	<0.001*			
Tumor location						
Ce,Ut	1.26	0.7-2.25	<0.001*			
Tumor depth						
pT3,4	3.89	2.24-6.75	<0.001*	2.12	1.15-3.88	0.015*
Lymph node metastasis						
N (+)	3.51	1.97-6.24	<0.001*	2.36	1.25-4.48	0.008*
Distant organ metastasis						
M (+)	2.31	0.98-5.4	0.054			
TLS number						
high	0.28	0.16-0.51	<0.001*	0.4	0.21-0.74	0.004*
CD8+T cell						
high	0.69	0.4-1.16	0.16			

Treg (FoxP3+)						
high	2.6	1.5-4.51	<0.001*	1.73	0.97-3.07	0.062
TAM (CD163+)						
high	1.97	1.16-3.34	0.012*			
FAP						
high	3.02	1.68-5.42	<0.001*			

Cox's proportional hazards model, AIC in a Stepwise Algorithm, statistical significance: P-value *<0.05, HR: Hazard ratio, CI: confidence interval, BMI: Body Mass Index

Progression-free survival

	Univariate			Multivariate		
	HR	95%CI	p-value	HR	95%CI	p-value
Age						
≧65	1.44	0.83-2.48	0.19			
Gender						
Male	5.69	1.38-23.4	0.016*	3.76	0.89-15.9	0.071
BMI						
<18.5	1.98	1.04-3.75	0.037*			
Neoadjuvant chemotherapy						
Yes	5.41	2.95-9.9	<0.001*	2.55	1.26-5.15	0.009*
Tumor location						
Ce,Ut	1.08	0.61-1.93	0.79			
Tumor depth						
pT3,4	4.35	2.55-7.44	<0.001*	2.01	1.06-3.82	0.033*
Lymph node metastasis						
N (+)	3.48	2.00-6.05	<0.001*	1.91	0.99-3.69	0.054
Distant organ metastasis						
M (+)	2.59	1.10-6.07	0.029*			
TLS number						
high	0.28	0.16-0.50	<0.001*	0.40	0.22-0.72	0.003*
CD8+T cell						
high	0.73	0.43-1.22	0.22	0.65	0.38-1.12	0.11
Treg (FoxP3+)						

high	2.96	1.71-5.12	<0.001*	1.84	1.00-3.38	0.050*
TAM (CD163+)						
high	2.28	1.36-3.84	0.002*			
FAP						
high	3.12	1.79-5.46	<0.001*			

Cox‘s proportional hazards model, AIC in a Stepwise Algorithm, statistical significance: P-value *<0.05, HR: Hazard ratio, CI: confidence interval, BMI: Body Mass Index
